# Supplementary figures and images for: A Fresh Look at Grape Powdery Mildew (Erysiphe necator) A and B Genotypes Revealed Frequent Mixed Infections and Only B Genotypes in Flag Shoot Samples
Source: Plants (Basel). 2020 Sep 7;9(9):1156. doi: 10.3390/plants9091156 (PMC7570353; doi:10.3390/plants9091156)

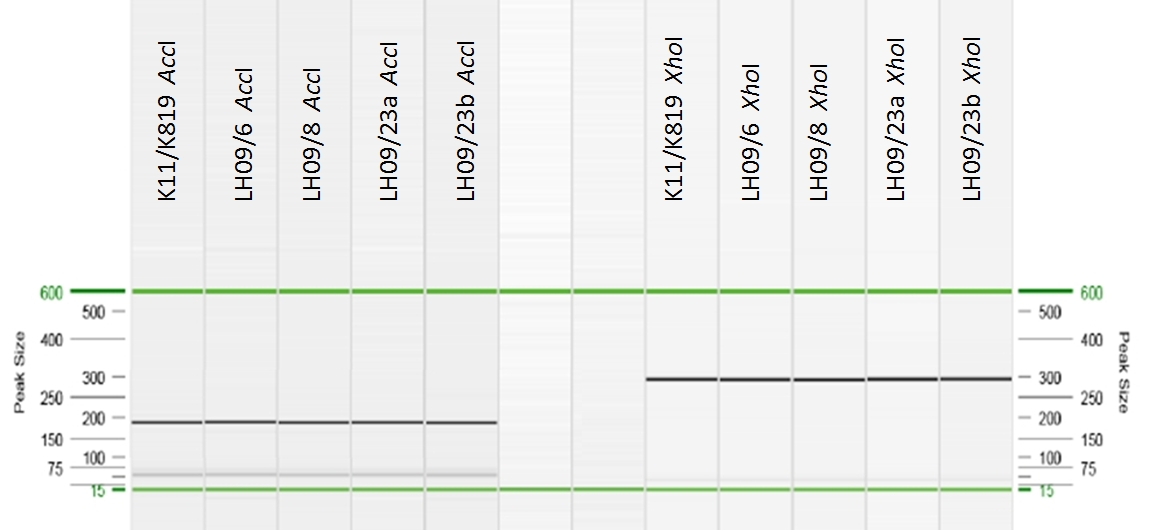

Supplement: Supplementary file 1 [file plants-09-01156-s001.zip › Supplementary Fig 1.jpg]
